# Toward implementation of combined incompatible and sterile insect techniques for mosquito control: Optimized chilling conditions for handling *Aedes albopictus* male adults prior to release

**DOI:** 10.1371/journal.pntd.0008561

**Published:** 2020-09-03

**Authors:** Dongjing Zhang, Zhiyong Xi, Yongjun Li, Xiaohua Wang, Hanano Yamada, Jieru Qiu, Yongkang Liang, Meichun Zhang, Yu Wu, Xiaoying Zheng

**Affiliations:** 1 Key Laboratory of Tropical Disease Control of the Ministry of Education, Sun Yat-sen University–Michigan State University Joint Center of Vector Control for Tropical Diseases, Zhongshan School of Medicine, Guangzhou, China; 2 Department of Microbiology and Molecular Genetics, Michigan State University, East Lansing, Michigan, United States of America; 3 Guangzhou Wolbaki Biotech, Guangzhou, China; 4 Lingnan Statistical Science Research Institute, Guangzhou University, Guangzhou, China; 5 Insect Pest Control Laboratory, Joint FAO/IAEA Division of Nuclear Techniques in Food and Agriculture, International Atomic Energy Agency, Vienna, Austria; 6 Department of Laboratory Medicine, Third Affiliated Hospital of Sun Yat-sen University, Guangzhou, China; Faculty of Science, Mahidol University, THAILAND

## Abstract

Combined incompatible and sterile insect technique (IIT-SIT) has been considered to be an effective and safe approach to control mosquito populations. Immobilization of male adults by chilling is a crucial process required for the packing, transportation and release of the mosquitoes during the implementation of IIT-SIT for mosquito control. In this study, effects of chilling on the *Aedes albopictus* males with triple *Wolbachia* infections (HC line), a powerful weapon to fight against the wild type *Ae*. *albopictus* population via IIT-SIT, were evaluated under both laboratory and field conditions. Irradiated HC (IHC) males were exposed to 1, 5 and 10°C for 1, 2, 3, 6 and 24 h. The survival rate of the post-chilled IHC males was then monitored. Longevity of post-chilled IHC males was compared to non-chilled males under laboratory and semi-field conditions. Mating competitiveness of IHC/HC males after exposure to 5 or 10°C for 0, 3 and 24 h was then evaluated. Effects of compaction and transportation under chilled conditions on the survival rate of IHC males were also monitored. The optimal chilling conditions for handling IHC males were temperatures between 5 and 10°C for a duration of less than 3 h with no negative impacts on survival rate, longevity and mating competitiveness when compared to non-chilled males. However, the overall quality of post-chilled IHC/HC males decreased when exposed to low temperatures for 24 h. Reduced survival was observed when IHC males were stored at 5°C under a compaction height of 8 cm. Transportation with chilling temperatures fluctuating from 8 to 12°C has no negative impact on the survival of IHC males. This study identified the optimal chilling temperature and duration for the handling and transportation of *Ae*. *albopictus* IHC male adults without any detrimental effect on their survival, longevity and mating competitiveness. Further studies are required to develop drone release systems specific for chilled mosquitoes to improve release efficiency, as well as to compare the population suppression efficiency between release of post-chilled and non-chilled males in the field.

## Author summary

Combining the incompatible insect technique (IIT) and sterile insect technique (SIT) is considered to be a promising approach to control *Aedes albopictus*, the world’s most invasive mosquito species which can transmit dengue and zika. The combined IIT-SIT technique depends on the release of overflooding numbers of mass-reared male mosquitoes to sterilize female mosquitoes through mating, resulting in the reduction of the wild mosquito population. In existing SIT programs against agricultural pests, male insects are often immobilized by chilling to pack, transport and release them and thereby increase handling efficacy. Chilling for the immobilization of male mosquitoes may also improve program efficiency in the IIT-SIT against mosquitoes. Thus, it is essential to ascertain the optimal chilling conditions for handling *Ae*. *albopictus* male mosquitoes. In this study, we found that *Ae*. *albopictus* males could be chilled at 10°C for 3 h without negative impacts on the biological qualities such as survival, longevity and mating competitiveness. Our results show that male mosquitoes could preserve a high level of quality under optimal chilling conditions, which indicates that immobilization by chilling is an important element for the combined IIT-SIT for mosquito control.

## Introduction

Mosquito-borne diseases, such as malaria, dengue, Zika, Chikungunya, Japanese encephalitis, West Nile and yellow fever, pose a serious threat to human health, causes millions of deaths every year [1]. Vector control using chemical insecticides remains the primary method of preventing most of the mosquito-borne diseases due to the lack of effective vaccines and drugs; however, the overuse of insecticides has numerous negative effects including the increase of insecticide resistance in mosquito populations, toxicity to non-target insects and animals, more than 200,000 human fatalities annually due to pesticide (e.g. organophosphorus) poisoning [2], as well as pollution of the environment. Thus, there is an urgent need to find other sustainable and environment-friendly solutions for mosquito control. The sterile insect technique (SIT) is one promising alternative. It is target-specific and can be combined with other vector-control tools as a component of an area-wide integrated pest management (AW-IPM) strategy [3]. The SIT is based on irradiation induced sterility of the released insects, and has been proven to successfully suppress or even eradicate several agricultural pests [3]. Based on the experience obtained from managing agricultural pests using the SIT, the Food and Agriculture Organization/International Atomic Energy Agency (FAO/IAEA) has been developing and transferring the SIT package for mosquito control and encouraging progress has been made in the past decade [4–8]. Simultaneously, a related technique, the incompatible insect technique (IIT) based on *Wolbachia*-induced sterility has also been tested and applied to control mosquitoes in recent years [9]. Due to the lack of a reliable and efficient sex separation method, the combined strategies with *Wolbachia* and irradiation can circumvent the associated risks of using only IIT or SIT for mosquito population control [10]. Until now, the combined strategy has been tested to control populations of several mosquito species including *Culex pipiens* [11], *Aedes polynesiensis* [12], *Ae*. *albopictus* [10] and *Ae*. *aegypti* [13]. A recent field study has shown that the combined IIT-SIT enabled a near elimination of *Ae*. *albopictus* populations in two isolated islands in Guangzhou, China [14].

The combined IIT-SIT strategy involves several steps including mosquito mass rearing, sex separation, irradiation, packing, transportation, release and monitoring. Progress has been achieved on mosquito mass rearing by developing standard operation procedures (SOPs) for egg handling (quantification, hatching and storage) [15–18], larval rearing (larval diet, rearing density, rearing conditions and rack system) [19–27] and adult rearing (rearing density, blood-feeding and cages) [28–30], respectively. The importance of sex separation for mosquito IIT-SIT is self-evident, and despite efforts and progress being made, there is still a lack of available genetic sexing strains for *Aedes* spp. [5]. However, a modified Fay-Morlan glass pupal sorters can be used to separate *Ae*. *albopictus* male and female pupae with a > 99.5% accuracy [14], and new automatic sex separation systems have been developed and tested to reduce labor and improve sorting efficiency [31]. Additionally, factors which might influence induced sterility during large-scale mosquito pupae irradiation procedures have been evaluated to further develop irradiation SOPs for achieving sufficient sterility [32] and reducing negative impacts of male quality so that they can compete successfully for wild females.

Aerial release of sterile males is another important implementation process for AW-IPM programs against mosquitoes. Unlike some agricultural pests, the limited flight ranges and fragile anatomy of *Aedes* mosquitoes [33] require the development of a unique release system. Unmanned Aircraft Systems (UAS, commonly known as drones) coupled with a specialized release mechanism have been developed to facilitate the continuous (or intermittent) aerial release of sterile adult tsetse flies (*Glossina* spp) [34] and codling moths (*Cydia pomonella*) [35], at a defined speed, interval, and density per surface area in the context of the SIT. This aerial release method has recently been adapted for immobilized and packed mosquitoes, showing high release efficiency and homogenous dispersal of the sterile males [36]. Prior to drone-based releases, sterile insects generally require packing and transportation. In SIT programs against agricultural pests, sterile insects are usually packed and transported in chilled conditions as the cold prevents them from moving. This not only facilitates the manual collection and packing, but also reduces physical damage to the insects when compacted [37–39]. However, chilling, and packing (compacting) induces stress which may lead to reductions of the biological quality of the sterile insects [40]. In addition, longer exposure durations to cold temperatures are likely to increase stress and therefore also damage caused to the insects [41]. Negative impacts on the overall quality of sterile males will affect their mating success, and thus the population suppression efficiency. It is therefore essential to determine the tolerable and optimal range of chilling temperature, duration, and compaction for male mosquitoes. Previous studies have indicated that chilling is suitable to handle *Anopheles arabiensis* and *Ae*. *aegypti* male adults if the handling conditions are optimized [42, 43].

This study focuses on the *Ae*. *albopictus* strain with a triple *Wolbachia* infection (known as the HC strain), a powerful weapon to fight against the wild type *Ae*. *albopictus* which is an important mosquito vector for several diseases such as dengue, Zika and Chikungunya, under the application of the combined IIT-SIT technique [14]. Effects of chilling temperature, duration and compaction on the resulting quality of male adults are evaluated in terms of survival, longevity and mating competitiveness. The results show that male mosquitoes could preserve a high level of quality under optimal chilling conditions, which indicates that immobilization by chilling is an important and useful element for the combined IIT-SIT strategies for mosquito control.

## Materials and methods

### Mosquito colonization

Two different *Wolbachia*-infected *Ae*. *albopictus* mosquito strains were used in this study: the HC strain with a triple *Wolbachia* infection (*w*AlbA, *w*AlbB and *w*Pip) and the GUA strain with a double infection (*w*AlbA and *w*AlbB). The HC strain was used for the chilling experiments while both HC and GUA strains were used for competitive mating experiments. Larvae were reared and fed daily on the diet (Wolbaki, Guangzhou, China) as previously described by Zhang et al. [24]. Pupae were collected and then sex separated using a modified Fay-Morlan glass sorter. Female and male pupae were placed inside 30 × 30 × 30 cm standard cages (stainless steel) at a sex ratio of 3:1 with continuous access to a 10% sugar solution. Adults were maintained in a climate-controlled room (rearing room) at 27 ± 1°C, 80 ± 10% RH, and a photoperiod of 12:12 h (L: D) and fed on ATP-supplemented blood [30].

### Pupal irradiation and adult chilling

Male pupae of the HC strain were collected and then irradiated using the X-ray irradiator (Wolbaki, Guangzhou, China) at a dose of 45 Gy [14]. The irradiated HC (IHC) male pupae were then allowed to emerge in stainless steel cages with access to a 10% sugar solution. Two- to three-day-old IHC male adults were chilled in refrigerators set up at different temperatures and for different durations to determine the optimal handling conditions for the transportation of IHC males.

### Effects of chilling on survival, knockdown and recovery time of IHC males

To determine the chilling temperature and duration threshold for IHC male adults, 70~130 males were randomly selected and transferred to petri dishes (diameter 10 cm × 1 cm) after immobilization at 5°C. The petri dishes (with covers) containing male adults were then stored in a refrigerator (Haier, China) set at either 1, 5 or 10°C for 1, 2, 3, 6 and 24 hours (h). After chilling, males were immediately transferred back to the above-mentioned rearing room and placed in an empty cage for recovery. Two hours later, the males that could not escape from the petri dishes were considered to be dead and their number was counted. Survival rate was calculated as: S_r_% = [100%—(the number of males that could not fly) / (the total number of post-chilled males) * 100%]. The temperature in the refrigerator was monitored by a Testo logger (175H1, Schwarzwald, Germany) and the results indicated that the fluctuation was approximately ± 1°C. Six replicates were performed for each treatment.

To verify the knockdown time for IHC male adults under the above-mentioned temperatures, a total number of 100~150 males were immobilized at 5°C and then quickly dispensed into several dishes (each dish containing 15–20 males). Males were allowed to recover at room temperature for over 30 mins and were then transferred to an incubator (SPX-80BE, Kuentian, China) set at 1, 5 or 10°C. Males were considered to have been knocked down by chilling if they were found to be laying down on their sides or on their backs when manually inspected through a glass window on the door of the incubator. The number of males knocked down was recorded every minute. After chilling for 3 or 24 h, 50–100 immobilized males were removed from the incubator and transferred to laboratory at 27 ± 1°C for recovery. Male adults were considered to have recovered once they were found to stand on their own or fly away. The number of recovered males was recorded every minute. Since very few males were found to recover after chilling at 1°C for 24 h, the recovery time was assessed at 1°C only for the 3 h treatment. The temperature in the incubator was monitored and verified by the Testo logger. Dead males were excluded from the recorded data in the knockdown and recovery experiment.

### Effects of chilling duration and feeding regimes on longevity of IHC males

Based on the above studies, we selected 5 and 10°C as the chilling temperatures to immobilize IHC males and the effects of chilling duration and feeding regimes on the longevity of post-chilled males were further assessed. Thirty IHC males, which had been chilled at 5 or 10°C for 3 or 24 h in the refrigerator (recorded as IHC_5°C-3h_/IHC_5°C-24h_ or IHC_10°C-3h_/IHC_10°C-24h_ males), were transferred to standard cages and maintained in the above-mentioned rearing room. Two different feeding regimes were applied daily to the post-chilled IHC males: either water only or sugar solution (10%). Mosquitoes were allowed to feed on water or sugar solution *ad libitum*. Mortality was recorded daily until all males had died. The non-chilled IHC (IHC_0h_) males were used as controls. Three replicates were performed for each treatment and control groups.

To predict the longevity of post-chilled IHC males in the field, we performed a series of Release-Recapture (RR) experiments in two semi-field rooms as shown in S1 Fig. Two to 3-day old IHC male mosquitoes were chilled at 5°C until they were immobilized. The immobilized male mosquitoes were then transferred on a piece of clean A4 paper. Approximately 8000 immobilized IHC males were poured from A4 paper and counted by weighing (1000 males ≈ 1.0 g) and placed in petri dishes (diam. × H: 120 × 10 mm). Two dishes were prepared and one was immediately taken to the semi-field room 1 (as control) and males released, while the other dish was maintained at 5°C for 3 h after which males were released in the semi-field room 2. Sugar solution at a concentration of 10% was continuously provided *ad libitum* to males for the duration of the experiment. Twenty-four hours post-release, two Biogents-Sentinel traps (Biogents AG, Regensburg, Germany) were used to recapture the males for 1 h daily for five continuous days. The recaptured males were frozen in the freezer and the number of recaptured males was recorded daily. The recapture rate was calculated as: Rr% = [(the total number of males recaptured) / 8000 * 100%]. The lethal time for 50% (LT50) IHC males was calculated based on the equation between the square–root transformed number of recaptured IHC males and post-release time. The RR experiments were separately repeated four times.

### Effects of chilling on the mating competitiveness and glucose level of IHC and HC males

We conducted two independent competitive mating experiments under laboratory conditions to assess the effects of chilling on the mating competitiveness index (*C*) of IHC and HC males [24]. In this study, the IHC males were chilled at 5°C for 0, 3 and 24 h (IHC_5°C-0h/3h/24h_) while HC males were chilled at 10°C for 0, 3 and 24 h (HC_10°C-0h/3h/24h_), respectively. Either post-chilled IHC or HC males (sterile males) competed with wild-type *Ae*. *albopictus* GUA strain males (fertile males) for copulation with GUA females. The competitive ratio was performed at 1:1 of sterile to fertile males (IHC/HC males: GUA males: GUA females = 1:1:1). Three or 5 replicates were conducted for each competitive treatment and three replicates for the sterile (IHC/HC males: GUA males: GUA females = 1:0:1) and fertile controls (IHC/HC males: GUA males: GUA females = 0:1:1). Since chilling did not affect the strength of cytoplasmic incompatibility (CI) induced by *Wolbachia*, the sterile controls set up in this competitive experiment were only for non-chilled IHC/HC males mated with GUA females.

For the competitive treatments, 50 sterile males were randomly selected and transferred to standard cages in the rearing room. Subsequently, 50 GUA males were added to the cages. Twenty-four hours later, 50 virgin GUA females (2–3 days old) were added to the cages for competitive copulation. For the controls, 50 IHC/HC males or 50 GUA males were mixed with 50 virgin GUA females, respectively. After two days of mating, males were removed with an aspirator and females were provided with blood-meals. Two days after blood feeding, an oviposition cup with a piece of moist filter paper was placed in the cages for collecting eggs for 3 days. Eggs were collected and dried for 7 days before hatching.

To evaluate the effects of chilling on the glucose level of *Ae*. *albopictus* male adults, IHC males (1 to 2 days old) were chilled at 5°C for 0, 3, 6, 12, and 24 h (IHC_5°C-0/3/6/12/24h_). Males were then transferred to the rearing room for recovery and then maintained in a cage continuously provided with a 10% sugar solution. Twenty-four hours later, 5 males were pooled into a 1.5 mL centrifuge tube as a single replicate and 12 replicates of biological assays were performed for each chilling duration. One stainless steel ball (diameter 3 mm) and 1 mL of distilled deionized water was added per tube. These samples were homogenized by a grinder (Jinxin, JXFSTPRP-32, Shanghai, China) set at 60 Hz for 30 sec. The homogenate was then aspirated with a syringe and passed through a 0.22 μm filter. The supernatant was then used for the glucose analysis. Glucose levels were determined by using the Glucose (GO) Assay Kit (SigmaAldrich, GAGO-20, USA) according to the manufacturer’s protocol with minor modifications. The above filtered homogenates were diluted 1:9 v/v with distilled deionized water and 50 μL of these diluted samples were then incubated with 100 μL assay reagent for 30 min at 37°C. After the incubation, 100 μL of 12N H_2_SO4 were added to stop the reaction. Then, the absorbance was measured at 540 nm by micropore detector (Sunrise, Tecan, Austria) and the average of glucose level for each male adult from each replicate was calculated from standard curve.

### Effects of packing on survival of IHC males

For improved efficiency, immobilized males need to be transported and packed under cold conditions. Therefore, in addition to chilling, the effects of packing on the survival rate of males should also be assessed. Two methods of packing were studied in this experiment: horizontal packing (slight compaction) and vertical packing (moderate compaction). For the horizontal packing treatment, IHC males immobilized at 5°C were estimated into batches of approximately 3000, 5000, 8000, and 10,000 males by weighing (1000 males ≈ 1.0 g), and were placed evenly in petri dishes (12 × 12 × 1 cm), with resulting densities of 20.8, 34.7, 55.6, and 69.4 males/cm^3^. After covering the filled petri dishes with lids, males were maintained at 5°C for 3 and 24 h, and at 10°C for 3 h. The horizontally packed height of males in the dishes was less than 1 cm. For vertical packing treatment, approximately 1700, 3400, 5100 and 6800 males were batched by weighing and then transferred to a 50 ml centrifuge tube, resulting in the vertical stacked height of males at 2, 4, 6 and 8 cm (or density at 174.7 males/cm^3^), respectively. The tube was vertically placed in the refrigerator at 5°C for 3 and 24 h, and at 10°C for 3 h. After chilling and storing, males were distributed evenly in a tray for recovery as described above. The data collected and the survival rate was calculated as mentioned above. Five replicates were performed for each treatment.

### Effects of transportation on the survival of IHC males

To test the effects of transportation on the survival rate of IHC males, approximately 10,000 immobilized males were placed in a petri dish (12 × 12 × 1 cm), which was then transferred to the center of a mobile incubator set at 10°C (± 2°C) and 80% RH. Five dishes were tested for each transportation test. The incubator was then driven by car for about 70 min to the field release site in Shazi Island, Guangzhou, China [14]. The dishes were taken out from the incubator and the lids were opened to allow males to fly out. During recovery, mosquitoes should avoid direct sunlight to reduce mortality due to the heat absorption (personal observation). The mean temperature in the field site was 30°C (± 2°C). Sixty minutes later, the lids were closed, and the dishes were brought back to the laboratory. The number of males which were unable to fly out was recorded. Dishes containing the same number of non-transported IHC males were used as controls to assess survival rate of males prior to transportation. Five replicates were performed for the transported males and three replicates for the non-transported males in each transportation experiment. The transportation experiments were repeated for three times.

### Data and statistical analysis

Data and statistical analyses were conducted using GraphPad Prism 6.0 software. For egg hatch rate and survival rate, data was arcsin-transformed before analysis. Dunnett’s multiple comparisons test or Two-sided Mann Whitney test was performed to compare the survival, mating competitiveness index and glucose level of IHC/HC males between non-chilled and chilled groups. One-way ANOVA analysis and Tukey *post-hoc* test was performed to compare the knockdown and recovery time of IHC males as well as the egg hatch rate in the mating competitive experiments. The longevity of IHC males with or without chilling was compared by Log-rank (Mantel-Cox) test. The number of daily recaptured males in each semi-field room was normalized by square–root transformation. Regression analysis was conducted between the transformed value and post-releases time. One-way ANOVA test was used to compare the slope of two regression equations. Two-sided Mann-Whitney test was used to compare the survival of males between non-transported and transported males. *P* < 0.05 indicated a significant difference.

## Results

### Effects of chilling on survival, knockdown and recovery time of IHC males

To optimize the chilling temperature for *Ae*. *albopictus* IHC male adults, we chilled male mosquitoes at different temperatures for different durations and then evaluated the survival, knockdown time, recovery time and longevity of post-chilled males. Compared to the control (without chilling, 0 h) group, no significant difference was observed on the survival rate of IHC males when they were chilled for less than 6 h at 5°C and 1 h at 10°C (Dunnett’s multiple comparisons test, *P* > 0.05), however, the survival was slightly reduced when the chilling time reached 24 h at 5°C and over 1 h at 10°C (Dunnett’s multiple comparisons test, *P* < 0.05) (Table 1). Reduced survival of males was observed when males were chilled at 1°C, regardless of the durations (Dunnett’s multiple comparisons test, *P* < 0.0001) (Table 1). After chilling for 24 h, the mean survival of IHC males was only 2.5% at 1°C as compared to about 89% at both 5 and 10°C (Table 1). When all the data was combined, it was found that IHC males chilled at 5 and 10°C had a higher survival than at 1°C (Tukey *post-hoc* test: *F*_(2, 87)_ = 35.07, *P* < 0.0001).

**Table 1:** Effects of chilling temperature and duration on survival rate, knockdown and recovery time of *Aedes albopictus* males.

Temperature (°C)	Duration (h)	Survival rate (%)	Adjusted *P* value	Knockdown time (min)	Recovery time (min)
Control	ND	96.2 ± 0.6	ND	ND	ND
1	1	89.7 ± 1.0	*P* < 0.0001	3.9 ± 0.2	ND
	2	84.3 ± 0.9	*P* < 0.0001		ND
	3	83.3 ± 0.7	*P* < 0.0001		59.1 ± 3.5
	6	63.1 ± 1.8	*P* < 0.0001		ND
	24	2.5 ± 0.9	*P* < 0.0001		ND
5	1	95.6 ± 0.7	*P* = 0.9889	7.4 ± 0.2	ND
	2	95.9 ± 0.5	*P* = 0.9992		ND
	3	95.3 ± 0.5	*P* = 0.8549		9.6 ± 0. 6
	6	93.6 ± 0.6	*P* = 0.0232		ND
	24	89.5 ± 0.5	*P* < 0.0001		33.9 ± 1. 7
10	1	95.8 ± 0.5	*P* = 0.9957	9.2 ± 0.4	ND
	2	93.3 ± 0.8	*P* = 0.0105		ND
	3	93.8 ± 0.7	*P* = 0.0412		1.5 ± 0.1
	6	93.0 ± 0.6	*P* = 0.0032		ND
	24	88.7 ± 0.5	*P* < 0.0001		1.4 ± 0.1

ND: none done.

All the data was presented as (Mean ± SEM).

Survival rate of post-chilled IHC males as compared to non-chilled males (control) by using the Dunnett’s multiple comparisons test (*P* < 0.05).

**Fig 1:**
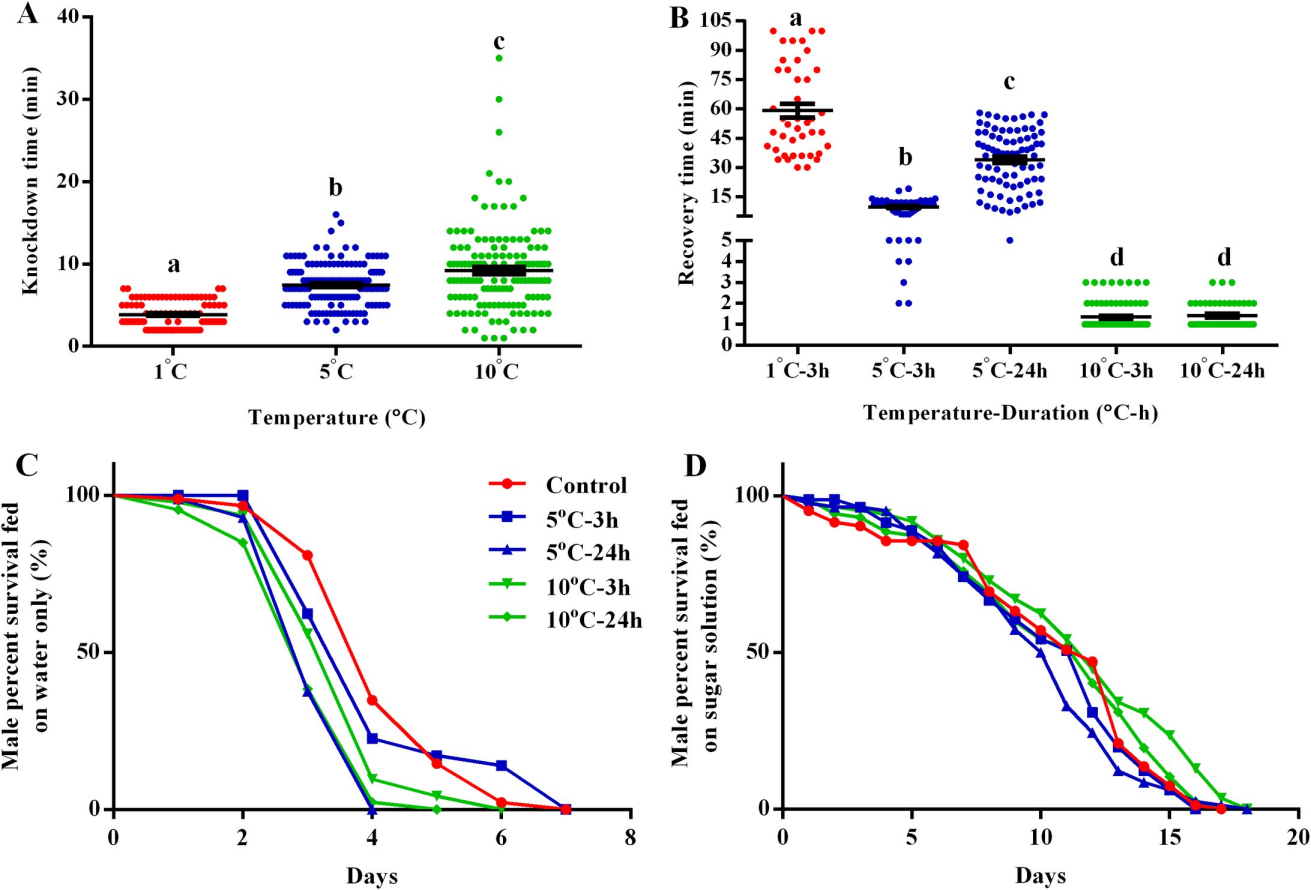
Knockdown time, recovery time and longevity of *Aedes albopictus* IHC males after chilling at varied temperatures and durations. A: Knockdown time of IHC males at 1, 5 and 10°C. B: Recovery time of IHC males after chilling at 1, 5 and 10°C for 3 or 24 h. C: Males were fed on water only after chilling. D: Males were fed on 10% sugar solution after chilling. Within Figures A and B, One-way ANOVA analysis and Tukey *post-hoc* test was performed to compare the knockdown and recovery time of IHC males when exposure to 1, 5 and 10°C for 3 or 24 h. Values followed by different lowercase letters were statistically different (*P* < 0.05). Each dot represents as individual male. Results are presented as (Mean ± SEM). Within Figures C and D, day number indicates time post-emergence. Kaplan-Meier curves were used to estimate the adult survival function.

The knockdown or recovery time for IHC males was associated with the chilling temperature. As shown in Fig 1A, a significant difference was observed on the knockdown time of IHC males chilled at these three temperatures (ANOVA and Tukey *post-hoc* test: *F*_(2, 375)_ = 62.06, *P* < 0.0001). Similarity, the recovery time for IHC males was significant different between chilling temperature and duration (ANOVA and Tukey *post-hoc* test: *F*_(4, 298)_ = 239.6, *P* < 0.0001) (Fig 1B). The mean time to knock down and recover IHC males with the three tested temperatures and two durations is shown in Table 1. Interestingly, we observed similar recovery times of IHC males when treated for 3 h and 24 h at 10°C, with a respective mean recovery time of 1.34 and 1.43 min. This mean time, however, was about 6- and 22-fold shorter compared to males chilled at 3 h and 24 h at 5°C, respectively (Table 1).

### Effects of chilling duration and feeding regimes on longevity of IHC males

The longevity of post-chilled IHC males was further assessed with two different feeding regimes under laboratory conditions. When post-chilled males were fed on water only, longevity of IHC males was reduced by a median of one-day when chilled for 24 h compared to the control (IHC_0h_) and those which were chilled at both 5 or 10°C for 3 h (IHC_5°C-3h_ and IHC_10°C-3h_) (Log-rank (Mentel-Cox) test: *χ*^*2*^ = 77.77, *df* = 4, *P* < 0.0001) (Fig 1C). The median adult longevity was 4 d for IHC_0h_, IHC_5°C-3h_ and IHC_10°C-3h_ males, and was reduced to 3 d for IHC_5°C-24h_ and IHC_10°C-24h_ males, respectively. However, when post-chilled males were fed on 10% sugar solution, IHC_5°C-24h_ males were found to live one-day less than other treatments of males (Log-rank (Mentel-Cox) test: *χ*^*2*^ = 16.79, *df* = 4, *P* = 0.0021) (Fig 1D). The median longevity was 11 d for IHC_5°C-24h_ males, and 12 d for the other treatment groups.

**Fig 2:**
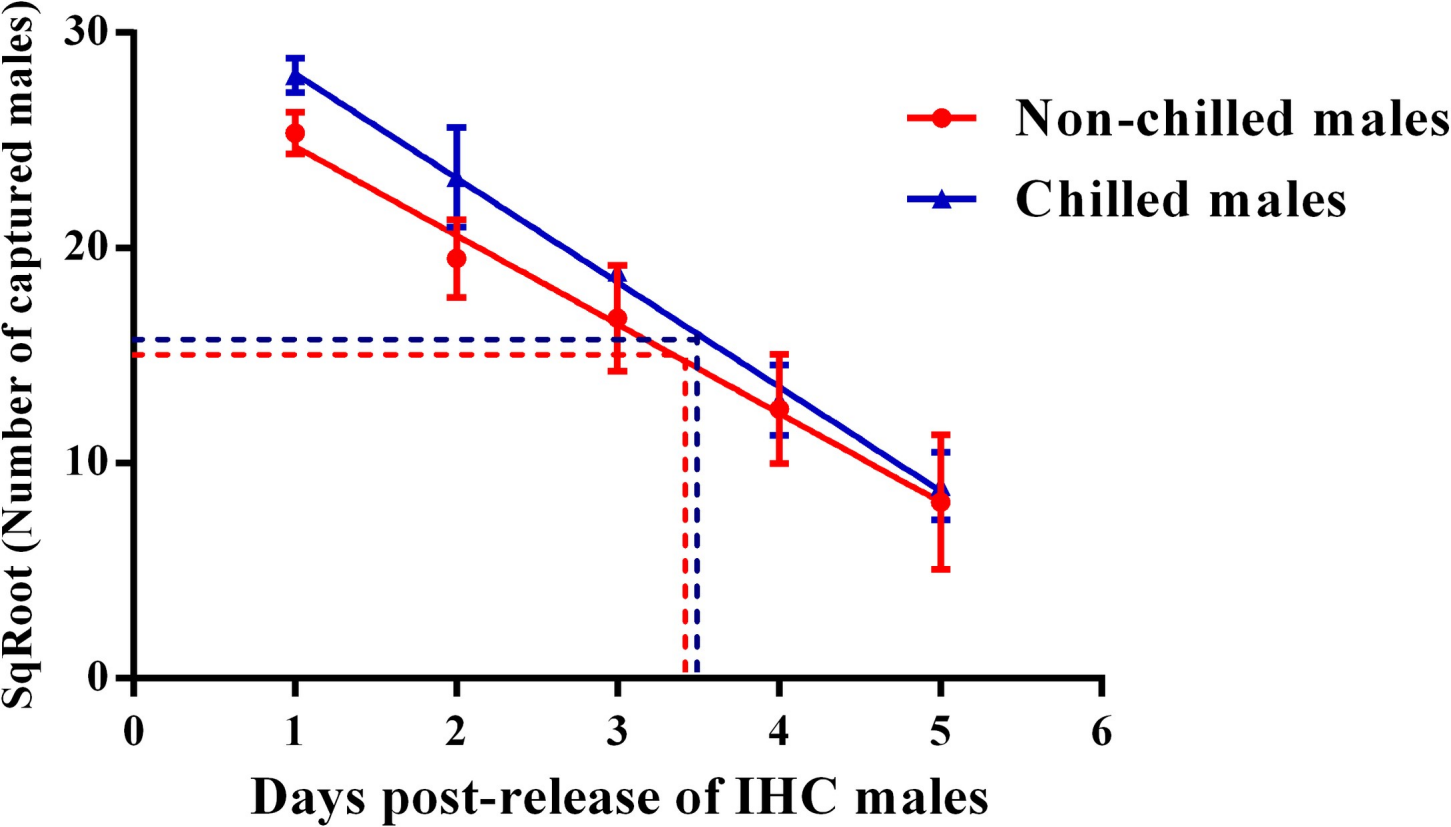
Linear relationship between the square–root transformed number of recaptured IHC males and post-release time. The red dotted line and blue dotted line represented the LT50 of non-chilled males and chilled males, respectively.

The longevity of post-chilled IHC_5°C-3h_ males was evaluated via a Release-Recapture experiment in a semi-field room. The release of non-chilled IHC males in a parallel room was used as a control. As shown in Table 2, the total recapture rate of IHC males within 5 days post-release was similar between the non-chilled and chilled groups (20.1% *vs* 24.5%). A liner relationship was observed between the daily square-root transformed number of IHC males recaptured and the post-release time in both semi-field rooms (Regression analysis: *P* < 0.0001) (Table 2 and Fig 2). When data from the 4 release repetitions was combined, the equation between the square–root transformed number of recaptured IHC males and post-release time was (y = -4.131 x + 28.85) for non-chilled males and (y = -4.850 x + 32.96) for post-chilled males (Fig 2). Based on the equation, the lethal time of 50% (LT50) of non-chilled IHC males was 3.49 days and 3.40 days for chilled males, respectively (Table 2). No significant difference was observed on the equation slopes between the non-chilled and chilled males (*F*_(1, 36)_ = 0.8078, *P* = 0.3747) (Table 2). The detailed information on LT50 of each release of IHC males is shown in S1 Table. With all data considered, our results indicate that chilling at 5 or 10°C for short periods has no impact on the survival and longevity of *Ae*. *albopictus* male adults under both laboratory and semi-field conditions.

**Table 2:** Comparison on the recapture rate and LT50 between non-chilled and post-chilled *Aedes albopictus* IHC males under semi-field condition.

Treatment	Replicate	No. of IHC males released	No. of IHC males recaptured	Recapture rate (%)	*P* value ^a^	LT50 (days) ^b^	*P* value ^c^
Non-chilled	4	8000	1606 ± 326	20.1 ± 4.1	*P* < 0.0001	3.49	*P* = 0.3747
Chilled	4	8000	1963 ± 194	24.5 ± 2.4	*P* < 0.0001	3.40	

^a^: A linear relationship was observed between the daily SqRoot transformed number of IHC males captured and post-releases time if the *P* value was lower than 0.05 using regression analysis.

^b^: LT50: Lethal time of 50% IHC males.

^c^: Slope of equation was not statistically different between the non-chilled and chilled males if the *P* value was higher than 0.05 using ANOVA analysis.

All the results were presented as (Mean ± SEM).

### Effects of chilling on the mating competitiveness and glucose level of IHC and HC males

**Fig 3:**
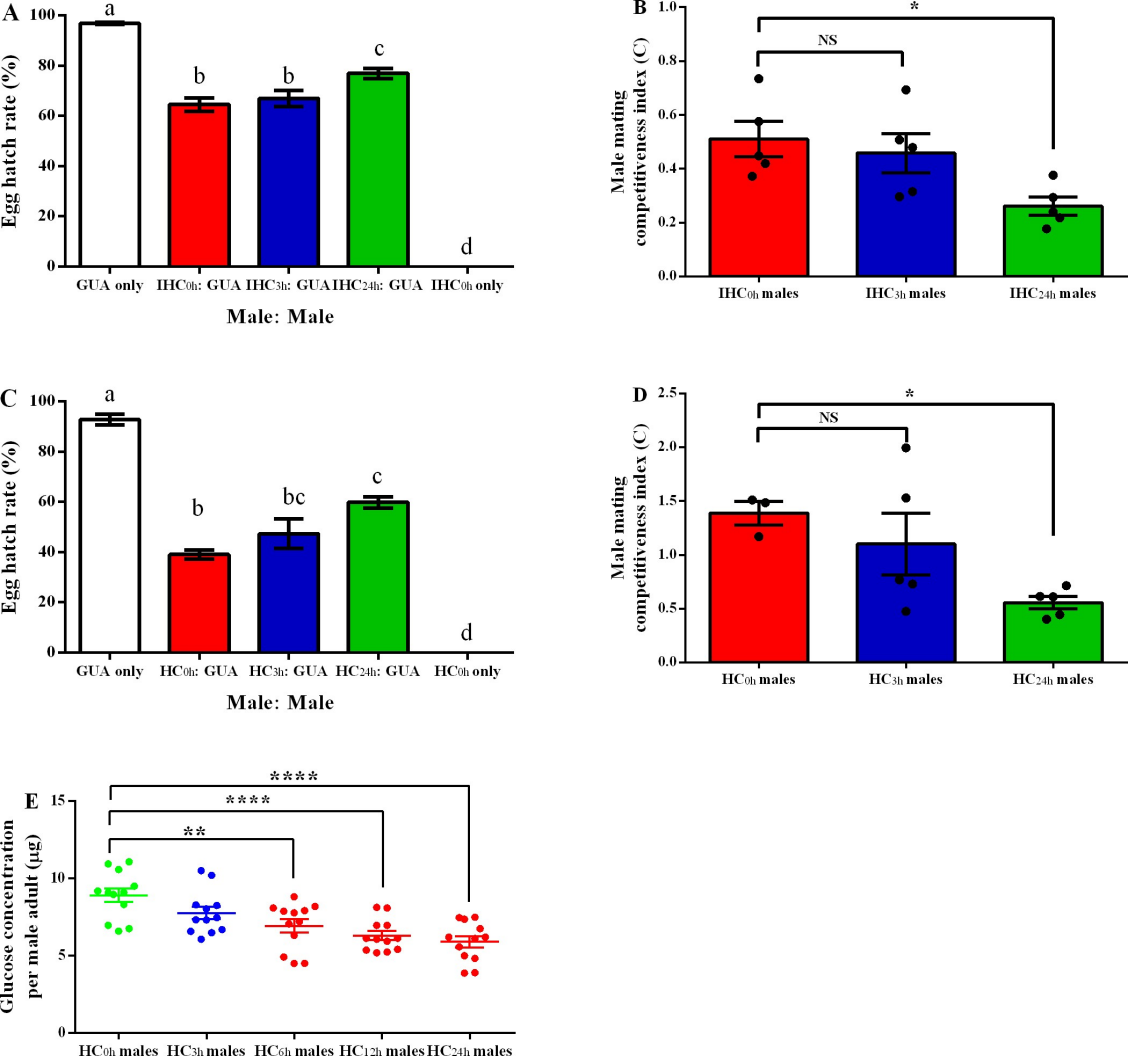
Effects of chilling on the male mating competitiveness and glucose concentration of *Aedes albopictus* IHC/HC male adults. A: Egg hatch rate from experimental cages containing a 1:1 ratio of sterile IHC_5°C-0/3/24h_ to fertile GUA males, or from control cages containing only sterile (IHC_5°C-0h_) or only fertile males. B: Male mating competitiveness of IHC_5°C-0/3/24h_ males. C: Egg hatch rate from experimental cages containing a 1:1 ratio of sterile HC_10°C-0/3/24h_ to fertile GUA males, or from control cages containing only sterile (HC_10°C-0h_) or only fertile males. D: Male mating competitiveness of HC_10°C-0/3/24h_ males. E: Glucose concentration of per IHC_5°C-0/3/6/12/24h_ male. Within A and C, data was arcsin-transformed and comparison was performed by ANOVA and Tukey *post-hoc* test. Different lowercase letters indicated the difference was significant (*P* < 0.05). Within B, D and E, Dunnett’s multiple comparisons test was used to compare the glucose levels between post-chilled IHC males and non-chilled males. NS indicated *P* > 0.05, ** indicated *P* < 0.01 and **** indicated *P* < 0.0001. Within A to E, results were presented as a Mean ± SEM.

To evaluate the mating competitiveness of males after chilling, we performed two separate competitive experiments between either post-chilled IHC males at 5°C or HC males (without irradiation) at 10°C, for 0, 3 and 24 h, and wild type males (GUA) for copulation with GUA females. Egg hatch rate significantly varied between different crosses for both two competitive experiments (ANOVA and Tukey *post-hoc* test, IHC_5°C_ males: *F*_(4, 16)_ = 131.8, *P* < 0.0001; HC_10°C_ males: *F*_(4, 14)_ = 63.14, *P* < 0.0001). The egg hatch rate of fertile controls from the two experiments were both higher than 90% while sterile controls had no eggs hatched, which indicated that irradiation had no added observable effect on sterility induction of males as complete sterility was both induced between either IHC_0h_ or HC_0h_ males and GUA females (Fig 3A and 3C).

Based on the egg hatch rate from the control and competitive cages, we calculated the *C* index of post-chilled IHC_5°C_ or HC_10°C_ males. As shown in Fig 3B, there was no statistical difference on *C* index between IHC_5°C-3h_ and IHC_5°C-0h_ males (Dunnett’s multiple comparisons test, *P* > 0.05), however, a decreased *C* index (~43% reduction) was observed in IHC_5°C-24h_ males when compared to IHC_5°C-0h_ males (Dunnett’s multiple comparisons test, *P* < 0.05). Similar results were obtained in the HC_10°C_ males, with a significantly decreased *C* index (~57% reduction) in HC_10°C-24h_ males (Dunnett’s multiple comparisons test, *P* < 0.05), but not in HC_10°C-3h_ males (Dunnett’s multiple comparisons test, *P* > 0.05), when compared to non-chilled males (Fig 3D). Due to the importance of energy preserved for the anti-chilling and mating of mosquitoes, we measured the glucose level of IHC male mosquitoes after chilling. As shown in Fig 3E, no significant difference was observed on the glucose levels between IHC_5°C-0h_ and IHC_5°C-3h_ males (Dunnett’s multiple comparisons test, *P* > 0.05), while the glucose levels of males which have been chilled for over 3 h was significantly decreased (Dunnett’s multiple comparisons test, 6 h: *P* < 0.01; 12 h: *P* < 0.0001; 24 h: *P* < 0.0001). An average of 51.3% reduction in glucose levels was observed in individual males which were chilled for 24 h compared to non-chilled male (5.89 μg *vs* 8.91μg). Taken together, our results indicate that a short duration (e.g. less than 3 h) of chilling at 5 and 10°C does not affect the mating competitiveness of IHC/HC males. In addition, the reduced mating competitiveness of males after chilling may associate with the reduced glucose level.

### Effects of packing on survival of IHC males

**Fig 4:**
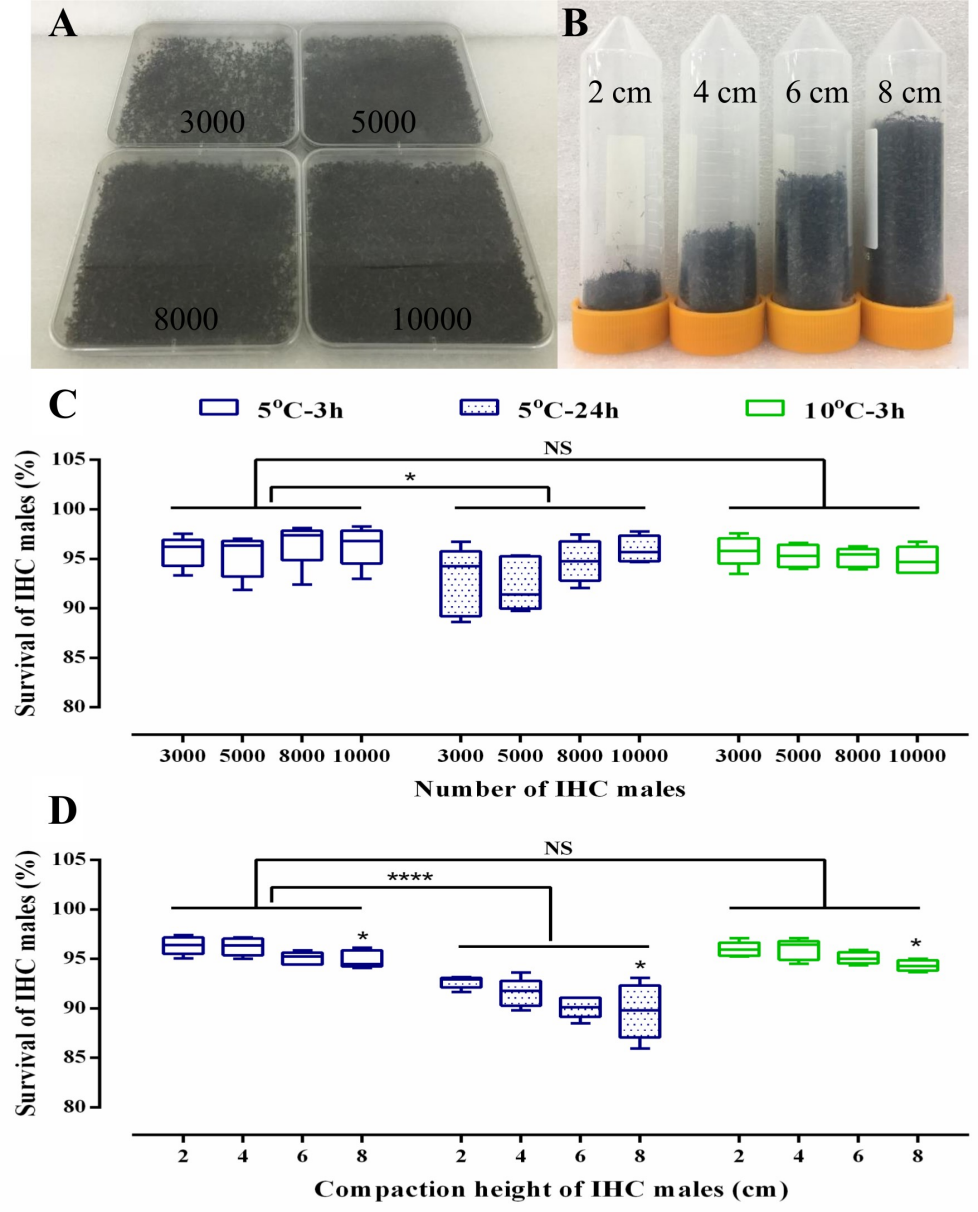
Survival of *Aedes albopictus* IHC males after chilling at 5 and 10°C when horizontally or vertically stacked. A: Number of IHC males chilled and horizontally stacked. B: Compaction height of IHC males when vertically stacked. C: Survival of IHC males horizontally chilled at 5 and 10°C for 3 h and 5°C for 24 h. D: Survival of IHC males vertically chilled at 5 and 10°C for 3 h and 5°C for 24 h. Within figures, One-way ANOVA analysis was performed to compare the effects of mosquito densities and compaction height on the survival of IHC males at the same chilling duration. At the same chilling temperature, survival of IHC males compacted at heights of 4, 6 and 8 cm was respectively compared to 2 cm by using Dunnett’s multiple comparisons test. Two-sided Mann-Whitney test was used to compare the survival of IHC males between 3 and 24 hours’ chilling. NS indicated *P* > 0.05, * represented for *P* < 0.05 and **** represented for *P* < 0.0001. Results were presented as Mean and the error bar indicates the minimum and maximum values.

Immobilized male mosquitoes are required to be transported under packed conditions for a period of time before field release. We therefore tested the effects of two different packing methods: horizontal (slight compaction, Fig 4A) and vertical (moderate compaction, Fig 4B) patterns, and assessed the impact on the survival of IHC males after chilling at 5°C for 3 and 24 h as well as at 10°C for 3 h. As shown in Fig 4C, no significant difference was observed on the survival of IHC males after chilling with the four tested densities of horizontal stacking (One-way ANOVA, 5°C-3 h: *F*_(3, 16)_ = 0.5636, *P* = 0.6468; 5°C-24 h: *F*_(3, 16)_ = 2.230, *P* = 0.1242; 10°C-3 h: *F*_(3, 16)_ = 0.5301, *P* = 0.6680). After the data from the same chilling temperature and duration were combined, we observed that a reduced survival of IHC_5°C-24h_ males compared to that of IHC_5°C-3h_ males (Two-sided Mann Whitney test: *U* = 108, *P* = 0.0121) while no significant difference was observed on survival between IHC_5°C-3h_ males and IHC_10°C-3h_ males (Two-sided Mann Whitney test: *U* = 134, *P* = 0.0758).

When males were compacted at different heights during chilling, we found that the compaction height had a significant impact on the survival of IHC males (One-way ANOVA, 5°C-3 h: *F*_(3, 16)_ = 4.043, *P* = 0.0257; 5°C-24 h: *F*_(3, 16)_ = 3.382, *P* = 0.0442; 10°C-3 h: *F*_(3, 16)_ = 5.074, *P* = 0.0117). Specifically, when compared to a vertical height of 2 cm, IHC males when compacted at heights of 4 cm and 6 cm had similar survival (Dunnett's multiple comparisons test, 5°C-3 h: *P* > 0.05; 5°C-24 h: *P* > 0.05; 10°C-3 h: *P* > 0.05), however, reduced survival was found for IHC males when compacted at a height of 8 cm (Dunnett's multiple comparisons test, 5°C-3 h: *P* < 0.05; 5°C-24 h: *P* < 0.05; 10°C-3 h: *P* < 0.05). When all data from the same chilling temperature and duration were combined, IHC_5°C-3h_ males showed a higher survival than IHC_5°C-24h_ males under vertical compaction condition (Two-sided Mann Whitney test: *U* = 0, *P* < 0.0001), while the former were not significantly different to IHC_10°C-3h_ males (Two-sided Mann Whitney test: *U* = 165.5, *P* = 0.3583) (Fig 4D). Our results indicate that 3 h of chilling at 5°C or 10°C with either horizontal stacking or vertical compaction height of less than 6 cm did not affect the survival of IHC males.

### Effects of transportation on the survival of IHC males

**Fig 5:**
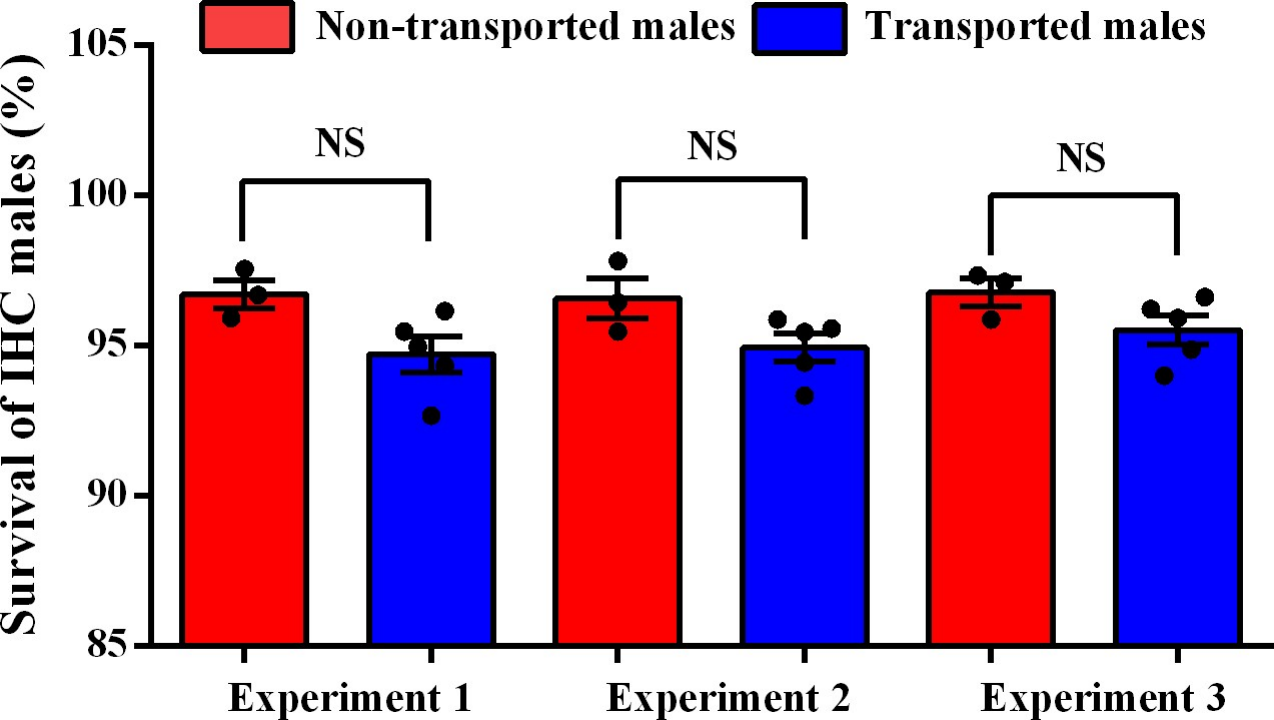
Survival of IHC males after transportation to the field release site. NS represented a non-significant difference according to the Two-sided Mann-Whitney test (*P* > 0.05). Results were presented as (Mean ± SEM).

It took approximately 1 h and 1.2 h to pack IHC male mosquitoes in the cold room, and transport mosquito from the mosquito factory to the field release site, respectively. The data from the temperature tracker showed that the temperature during packing and transportation fluctuated between 8 and 12°C. As shown in Fig 5, a slight reduction of survival of the IHC males was observed after transportation following the three independent experiments, however, the reduction was not statistically significant between non-transported and transported males (Two-sided Mann-Whitney test, Exp. 1, *P* = 0.0714; Exp. 2, *P* = 0.1429; Exp. 3, *P* = 0.2500). An average of 95.1% of the IHC males survived after the three transportation experiments. While the average survival of post-packed IHC males without transportation was 96.70% (96.58% ~ 96.79%).

## Discussion

Cold tolerance, the ability of an insect to survive under low temperature conditions, is one of the key factors for the distribution and survival of insects in the field [44]. Generally, insects will stop moving to reduce metabolism rates to protect themselves from chilling. Therefore, when applying the SIT to manage agricultural pests, the sterile males are usually immobilized by chilling during packing, transportation and release, thereby reducing the insects’ activity and mutual injuries, and thus allowing the insects to be compacted at high densities and improving handling efficacy. *Ae*. *albopictus* is considered to be one of the most invasive species in the last 3–4 decades [45] and for its successful invasion, especially for spatial expansion to cooler climates, this species has adapted to cope with cooler temperatures [46]. This indicates that chilling is also potentially suitable for the packing, transportation, and release of *Ae*. *albopictus* under the application of combined IIT-SIT for mosquito control. Therefore, we studied the effects of chilling on several key biological parameters correlated to the quality of *Ae*. *albopictus* HC strain male mosquitoes in the aim to improve the efficiency of mosquito packing, transportation and release. Our results indicate that a temperature range of 5–10°C is optimal for the immobilization of male adult *Ae*. *albopictus* with minimal effect observed on the survival rate of post-chilled males. In addition, a 3-hour chilling duration at 5–10°C did not influence the longevity and mating competitiveness of males, regardless whether they were exposed to irradiation or not at pupal stage. Moreover, there was no influence on survival rates of males after chilling at 5–10°C for 3 h under horizontal stacking conditions, however, a decreased survival rate was observed when males were vertically compacted at heights of 8 cm. Lastly, no negative impact on the survival of males was found post transportation. Our study confirmed that chilling was an appropriate way to immobilize male mosquitoes without negative impacts on the qualities such as survival and competitiveness of males if they were handled under optimized chilling conditions.

It is the general consensus to release adult mosquitoes instead of pupae in mosquito SIT programs. Before release, immobilized male adults are packed and then transported under chilled conditions. However, studies on the tsetse fly (*Glossina* spp) and Mediterranean fruit fly (*Ceratitis capitata*) have shown that biological qualities (e.g. longevity, flight ability and mating abilities, etc) of post-chilled males can decrease if they were not handled under optimized conditions [39, 47]. Our results are consistent with the previous reports that *An*. *arabiensis* male adults could be exposed to temperatures ranging from 4–10°C for up to 24 h without affecting longevity, while reduced longevity was apparent when exposed to 2°C [42]. Post-chilled *Ae*. *aegypti* males exposed to 8 or 10°C lived longer than those exposed to 4°C and below [48]. In addition, decreased flight ability and mating propensity of *Ae*. *aegypti* males were observed after chilling below 8°C for up to 2 h. In our study, we found that the optimal chilling temperature for *Ae*. *albopictus* males ranges between 5 and 10°C with minimal effects on post-chilled male survival rate. On the contrary, survival rate significantly decreased when males were exposed to 1°C. Chilling temperature at 5 or 10°C for up to 3 h did not influence the longevity of chilled males, however, male longevity significantly decreased if the chilling duration extended to 24 h. This indicates that the optimized chilling conditions, e.g. temperature and duration, were different for each insect species. Interestingly we found that a very short recovery time was required for male mosquitoes after chilling at 10°C, even for a duration of up to 24 h. Generally, the shorter the recovery time required, the better for the immobilized male mosquitoes after release, given that a short time of males laying on the ground before flying will reduce the probability of predation and other causes of mortality. Field temperature is another key factor that should be considered prior to the release of chilled male mosquitoes. The optimal temperature ranges in field are between 23 to 30°C (personal observation). Lower temperature will affect the recovery time and flight ability of male mosquitoes while higher temperatures will cause excess mosquito morality. Therefore, the release time should be adjusted to meet the required release temperature according to the season. For example, releases can be performed at noon in early spring or late autumn as the temperature is relatively low in the morning. On the contrary, releases in the morning or sun-set can be planned if temperatures are too high at noon.

Mating competitiveness of sterile males is an important parameter for the mosquito IIT-SIT approach. All steps required for the implementation of the technique will, to some extent, affect the males’ mating competitiveness. Effects of *Wolbachia w*Pip transinfection [49], and/or irradiation [50] as well as mass rearing [24] on the mating competitiveness of *Ae*. *albopictus* males have been previously studied. In this study, we further tested the impacts of chilling on the mating competitiveness of *Ae*. *albopictus* HC males and the results showed that the chilled males at 5 or 10°C for up to 3 h, with or without irradiation, exhibited equal mating competitive performance compared to non-chilled males (Fig 3B & 3D). This indicates that a short-time exposure to low temperatures has little to no impact on the mosquito mating ability. It has also been shown that a short-time (< 5 h) exposure of adult female *Ae*. *albopictus* to a low subzero temperature (-9°C) in water either before or after a blood meal had no detrimental impact on the fitness costs of these adults [51]. The increased glycerol and glucose levels as well as the upregulation of heat shock protein 70 (Hsp70) in adult female *Ae*. *albopictus* was considered to be involved in mosquitoes’ mechanism to cope with cold temperature [51]. However, the competitive performance of the males in this study was reduced by approximately 50% when the chilling duration reached 24 h (Fig 3B & 3D). This was similar to a previous study showing that long-time exposure to low temperatures reduces the mating competitiveness of sterile males [39]. The decline in mating competitiveness may be due to physiological, or cell damage in male mosquitoes which can be caused by prolonged low temperature [43], resulting in a decrease in sperm number or activity, thereby reducing the insemination rate.

Sugars are one of several important cryoprotectants in insects to minimize or eliminate freeze damage [52]. A previous study has indicated that the accumulation of glucose in adult *Ae*. *albopictus* is important during recovery from cold exposure but not during cold exposure itself [51]. However, in this study we observed the decreased glucose levels in the post-chilled male mosquitoes. Those mosquitoes chilled for 24 h at 5°C resulted in a 51.3% reduction in glucose levels when compared to non-chilled males (Fig 3E). The two studies show contradicting results on glucose levels of *Ae*. *albopictus* mosquitoes after cold exposure, and the differing results could be attributed to the timing at which the glucose levels of mosquitoes were measured. The former study selects mosquitoes 1 to 4 h post cold exposure. There is no doubt that energy is required to immediately accumulate in mosquitoes during recovery [51]; however, 24 h post-chilled males were selected to measure the glucose content in this study. Therefore, there is possibility that the initial glucose levels increase in male mosquitoes during recovery after cold exposure but gradually decrease when they have completely recovered, but which requires further investigation. As glucose plays an important role in maintaining life, flight and mating of insects [53], we speculate that the decline in mating competitiveness may also be linked to the decrease in glucose reserves in post-chilled male mosquitoes as observed in this study. Until recently, assessments of male mosquito biological quality have involved time-consuming and tedious methods (such as longevity and competitiveness studies). More recently, a novel flight test device (FTD) which provides results within 2 h has been developed for measuring the flight ability of males following various treatments. The propensity to fly is an important parameter for indicating the quality of (sterile) males [48]. In addition to such tests, we propose glucose measurement as an additional, simple and effective method to detect the qualities of sterile males after chilling.

Immobilized male mosquitoes can be packed in a container with a chilling system and then transported to the target area for release. Therefore, two stacking patterns on the survival of male mosquitoes were tested in this study. The results indicated that horizontal stacking at a density of 69.4 males per cm^3^ did not affect the survival of post-chilled males. In addition, we observed that the longevity of post-chilled males at 5°C for 3 h at the above tested density was similar to that of normal males under semi-field conditions. On the contrary, a reduced survival rate was observed when males were vertically compacted a height of 8 cm. This confirms previous studies in which reduced qualities were observed in *Ae*. *aegypti* males which were compacted with a weight of more than 5 grams [48], as well as in Mediterranean fruit flies post-chilled at 4°C for 3 h packed in high densities [39]. Thus, our results have emphasized the importance of compaction height of immobilized male mosquitoes. Interestingly, we found the “locking legs” phenomenon of mosquitoes did not affect the survival of males as almost all of the males could untangle their legs before flying away. We believe that it is important to understand the effects of horizontal or vertical stacking on chilled males as these two patterns can be applied to different release methods: the former for ground release (fixed point release) and the latter for aerial release (continuous release). In addition, space requirements for transportation is greatly reduced if males are immobilized and compacted. A comparison of non-chilled and chilled conditions for the transportation of one million *Ae*. *albopictus* male adults is shown in S2 Table.

In conclusion, we recommend the chilling of *Ae*. *albopictus* male mosquitoes at 10°C for less than 3 h due to its lack of negative effects on their survival, longevity and mating competitiveness. We selected 10°C since the post-chilled males could recover in a shorter time at room temperature. Further studies will focus on increasing the chilling duration for male mosquitoes without affecting the quality. The extension of the chilling duration not only provides more flexibility for operations such as mosquito packing, transportation and field release, but also increase distance from the mosquito factory to the target area. For control areas located long distances away from the mosquito factory, it is conceivable to transport mosquitoes at pupal stage under chilled conditions by air, but this would still require the establishment of an emergence room as well as a cold room around in the vicinity of the release area. More studies are required to test the feasibility of transporting mosquito pupae under low temperatures. In addition, future studies should focus on the flight ability, life span, and mating competitiveness of the chilled male mosquitoes in the field. For improving release efficiency, aerial release of sterile male adults is required in SIT in operational scale, and our results can be further applied for the design of suitable drone- and other release systems for mosquitoes and thereby enhancing the mosquito population suppression efficacy with the combined IIT-SIT strategy.

## Supporting information

S1 Table Information on the LT50 of each release of *Aedes albopictus* IHC males with or without chilling under semi-field condition. (DOCX)

S2 Table Comparison between non-chilling and chilling ways for transportation one million *Aedes albopictus* HC males. (DOCX)

S1 Fig Layout of semi-field rooms for release-recapture experiments. The size of each room was about 48 square meters (8 meters in length and 6 meters in width). There was stainless steel mesh in the windows, which guaranteed the same conditions between the two rooms and the field. The mesh was also used to prevent the escape of male mosquitoes from the room to the field during experiments. Green plants were placed in the rooms (in the green area) as a habitat for the released males. Two Biogents-Sentinel traps were placed in each room for recapturing males after release. The red point indicates the release position. There was a buffer zone in the entrance of the semi-field rooms to prevent the escape of males.
(TIF)

S1 Text Supporting methods. Standard operation procedures for packing and releasing sterile *Aedes albopictus* male mosquitoes.
(DOCX)

S1 Data Excel spreadsheet containing, in separate sheets, the underlying numerical data and statistical analysis for Tables and Figures. (XLSX)
