# Supplementary material for: Toward implementation of combined incompatible and sterile insect techniques for mosquito control: Optimized chilling conditions for handling Aedes albopictus male adults prior to release
Source: PLoS Negl Trop Dis. 2020 Sep 3;14(9):e0008561. doi: 10.1371/journal.pntd.0008561 (PMC7470329; doi:10.1371/journal.pntd.0008561)
Supplement: S1 Table — (DOCX) [file pntd.0008561.s001.docx]

Table S1 Information on the LT50 of each release of *Aedes albopictus* IHC males with or without chilling under semi-field condition

| Male release | Treatment | SqRoot (Number of males recaptured) | | | | | Liner relationships ^a^ | LT50 (d) |
| --- | --- | --- | --- | --- | --- | --- | --- | --- |
|  |  | Day 1 | Day 2 | Day 3 | Day 4 | Day 5 |  |  |
| 1^st^ Release | Non-chilling | 27.51 | 24.14953 | 22.48555 | 19.34942 | 16.22344 | y = -2.737*x + 30.16 | 5.51 |
|  | Chilling | 28.84441 | 28.12828 | 18.43909 | 17.82133 | 11.93315 | y = -4.413*x + 34.27 | 3.88 |
| 2^nd^ Release | Non-chilling | 26.43861 | 20.19901 | 17.60682 | 12.52996 | 9.949874 | y = -4.065*x + 29.54 | 3.63 |
|  | Chilling | 29.37686 | 25.72936 | 18.97367 | 11.35782 | 11.35782 | y = -5.041*x + 34.48 | 3.42 |
| 3^rd^ Release | Non-chilling | 23.45208 | 15.74802 | 10.48809 | 7.211102 | 2.44949 | y = -5.054*x + 27.03 | 2.67 |
|  | Chilling | 28.03569 | 17.60682 | 18.9473 | 10.90871 | 6.708204 | y = -4.935*x + 31.25 | 3.17 |
| 4^th^ Release | Non-chilling | 23.97777 | 17.94807 | 16.32993 | 10.97877 | 4.131182 | y = -4.666*x + 28.67 | 3.07 |
|  | Chilling | 25.77854 | 21.63947 | 19.18333 | 11.5931 | 5.750362 | y = -5.010*x + 31.82 | 3.18 |

a: y represented for the SqRoot transformed number of males recaptured; x represented for the days post release of males.

LT50: Lethal time of 50% released males.
