# Supplementary material for: Toward implementation of combined incompatible and sterile insect techniques for mosquito control: Optimized chilling conditions for handling Aedes albopictus male adults prior to release
Source: PLoS Negl Trop Dis. 2020 Sep 3;14(9):e0008561. doi: 10.1371/journal.pntd.0008561 (PMC7470329; doi:10.1371/journal.pntd.0008561)
Supplement: S2 Table — (DOCX) [file pntd.0008561.s002.docx]

Table S2 Comparison between non-chilling and chilling ways for transportation one million *Aedes albopictus* HC males

| Parameter | Mosquito holders | | |
| --- | --- | --- | --- |
|  | Release bucket | Release cage | Release box |
| Chilling system for package and transportation | No | No | Yes |
| Dimensions (cm) (L * W * H) | 17 diameter × 17 | 30 × 30 × 30 | 12 × 12 × 1 |
| Volume (cm^3^) | 3857 | 27000 | 144 |
| Number of male adults held per unit | 800~1000 | 8000-10000 | 10000-12000 |
| Male adult status | Active | Active | Anesthetized |
| Density (number of mosquitoes/cm^3^) | 0.21~0.26 | 0.30~0.37 | 69.4~83.3 |
| Number of units required for one million males | 1000~1250 | 100~125 | 84~100 |
| Space for transportation one million males (m^3^) | 3.85~4.76 | 2.70~3.33 | 0.012~0.014 |
| Labor-Handling | Heavy | Heavy | Slight |
| Labor-Releasing | Heavy | Heavy | Slight |
| Labor-Cleaning | Heavy | Heavy | Slight |
| Application | Small scale | Small/Medium scale | Medium/Large scale |
