# Supplementary figures and images for: Toward implementation of combined incompatible and sterile insect techniques for mosquito control: Optimized chilling conditions for handling Aedes albopictus male adults prior to release
Source: PLoS Negl Trop Dis. 2020 Sep 3;14(9):e0008561. doi: 10.1371/journal.pntd.0008561 (PMC7470329; doi:10.1371/journal.pntd.0008561)

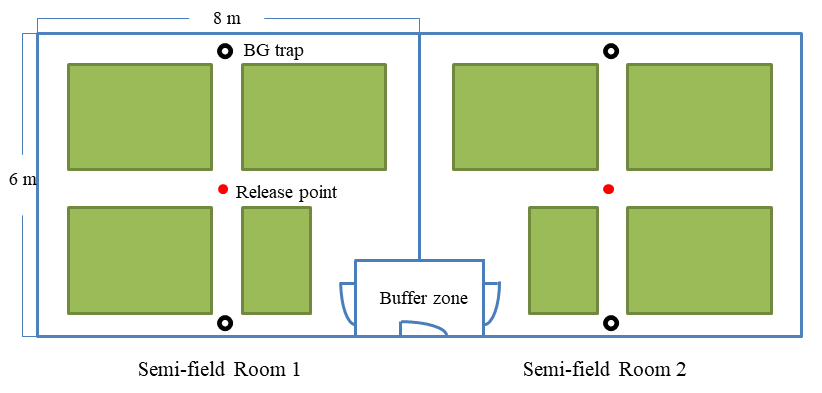

Supplement: S1 Fig — The size of each room was about 48 square meters (8 meters in length and 6 meters in width). There was stainless steel mesh in the windows, which guaranteed the same conditions between the two rooms and the field. The mesh was also used to prevent the escape of male mosquitoes from the room to the field during experiments. Green plants were placed in the rooms (in the green area) as a habitat for the released males. Two Biogents-Sentinel traps were placed in each room for recapturing males after release. The red point indicates the release position. There was a buffer zone in the entrance of the semi-field rooms to prevent the escape of males. (TIF) [file pntd.0008561.s003.tif]
