# Supplementary material for: Toward implementation of combined incompatible and sterile insect techniques for mosquito control: Optimized chilling conditions for handling Aedes albopictus male adults prior to release
Source: PLoS Negl Trop Dis. 2020 Sep 3;14(9):e0008561. doi: 10.1371/journal.pntd.0008561 (PMC7470329; doi:10.1371/journal.pntd.0008561)
Supplement: S1 Text — Standard operation procedures for packing and releasing sterile Aedes albopictus male mosquitoes. (DOCX) [file pntd.0008561.s004.docx]

**Supporting method**

**Standard operating procedures for packing and releasing sterile *Aedes albopictus* male mosquitoes**

1. Transfer cages containing 2-3-day old male mosquitoes to a cold room (or incubator) set at 10 ℃ for immobilization.

2. After 30 minutes, carefully pour all of the male mosquitoes out of the cages and into a container for measuring the total weight of the mosquitoes.

3. Randomly collect approximately 1 gram of immobilized male mosquitoes and count the number of males. Repeat this step 3 times. Count and record the number of female mosquitoes detected in each 1 gram of the and calculate the female contamination rate (FCR).

*Males are approved for release if the average FCR value from three independent samples are below 0.3%.

4. Aliquot immobilized males into petri dishes (12 × 12 × 1 cm) with each dish containing approx. 10000 males (Fig. 1). Cover the dishes with lids.

5. Transfer the filled dishes to the mobile incubator (Figs. 2 and 3) in the cold room, and stack the dishes inside. The temperature of the incubator is set at 10 ℃. Place damp cotton in the incubator to increase the humidity during transportation. Ice bags can be suppled if needed, such as when outer temperature is too high.

6. At the release site, take the dishes out of the incubator and open the lids to allow males to fly out (Fig. 4). Where possible, perform the releases under trees or in the shade to reduce the molarity of male mosquitoes during their recovery. Walking while release can help to improve the dispersal of males.

Generally, expect to see more then 95% of males recover and fly away within 10 minutes. The remaining males are discarded as they are either dead or damaged.


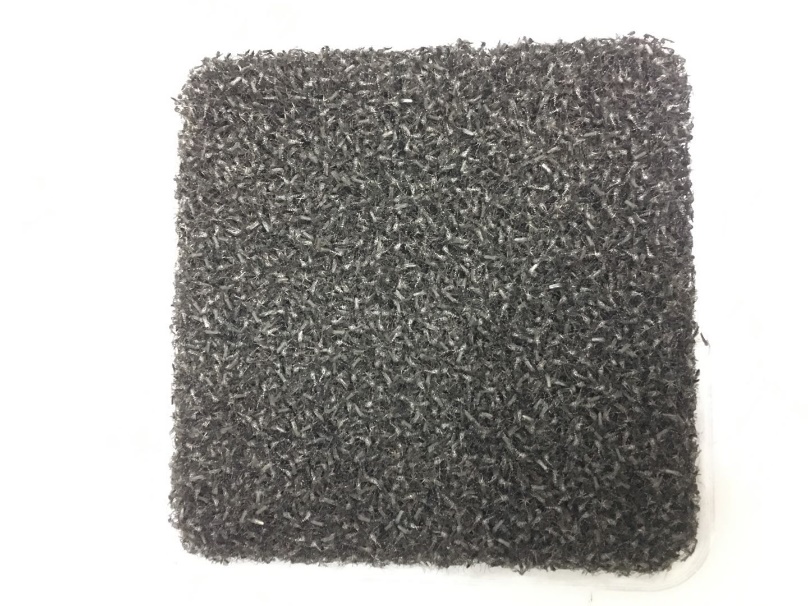


Fig. 1 Immobilized male mosquitoes are aliquoted into a petri dish


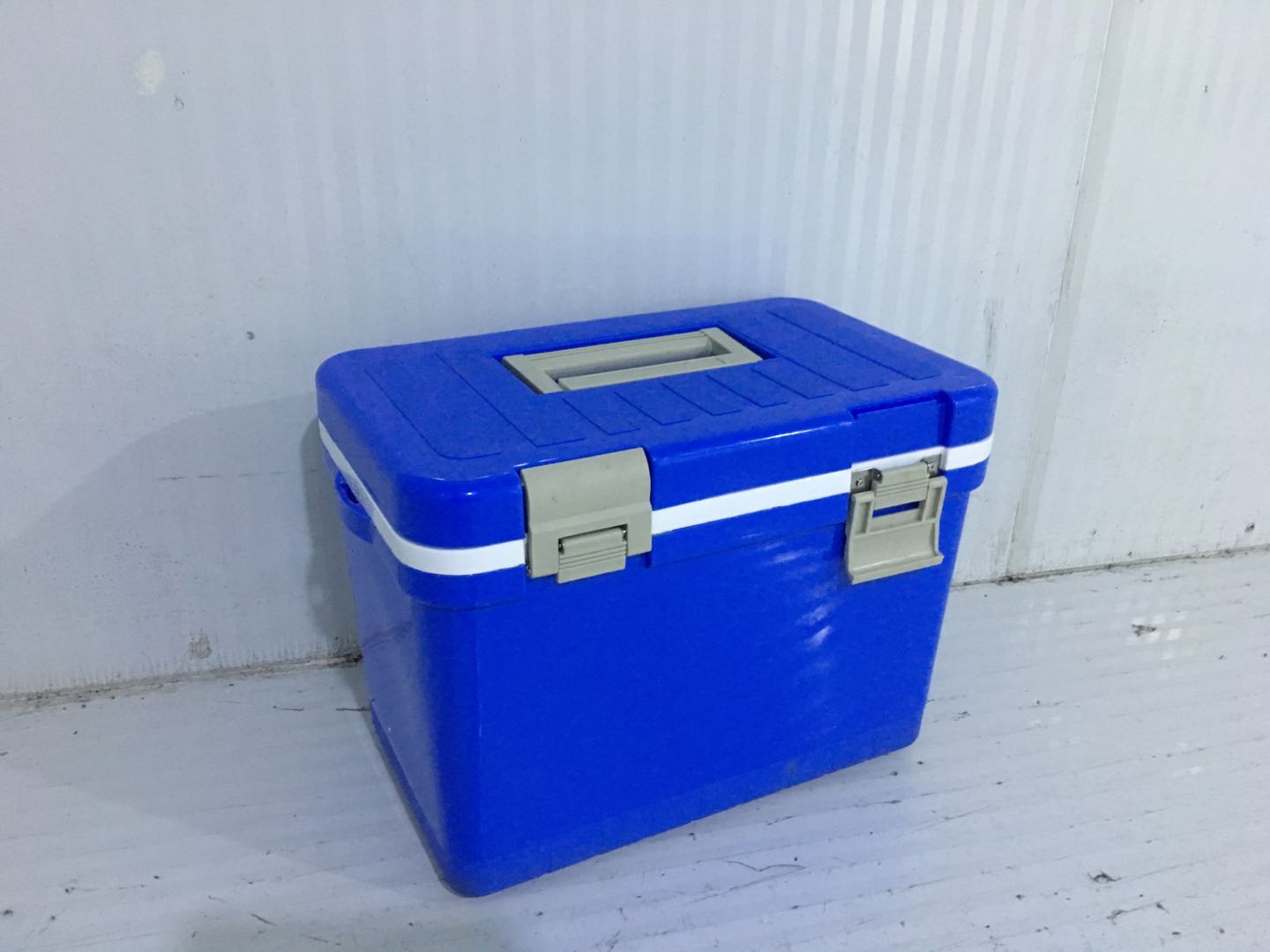


Fig. 2 Mobile incubator


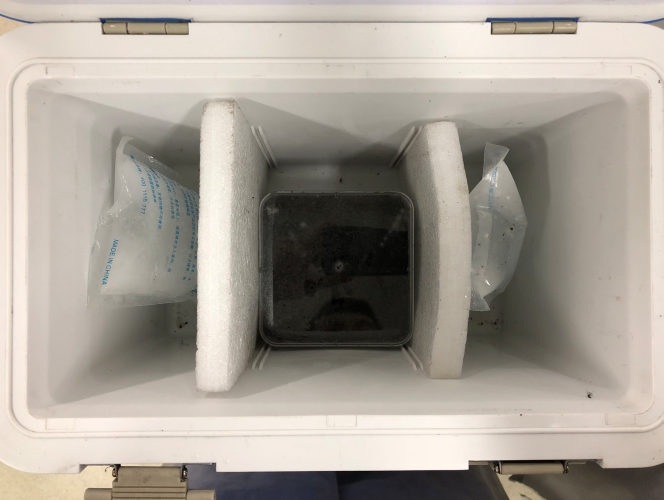


Fig. 3 Vertically stacked petri dishes inside the mobile incubator


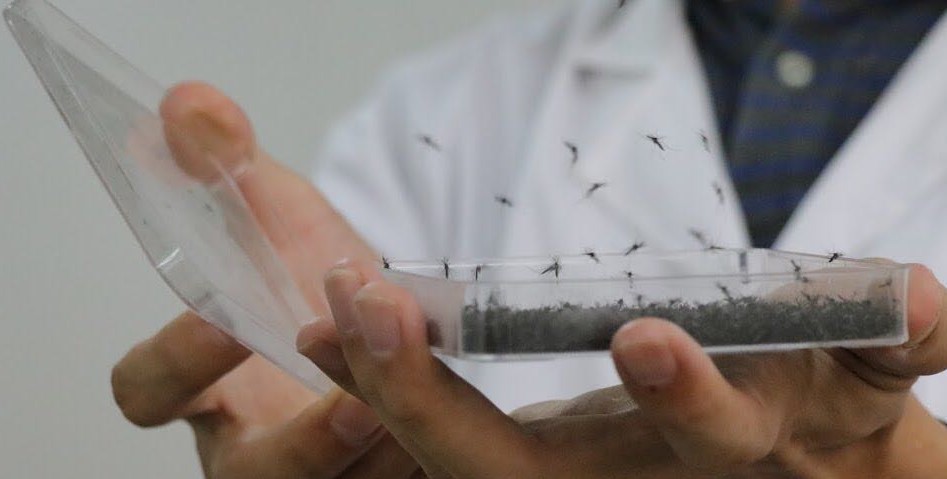


Fig. 4 Recovery of chilled male mosquitoes
